# Room-temperature polarized spin-photon interface based on a semiconductor nanodisk-in-nanopillar structure driven by few defects

**DOI:** 10.1038/s41467-018-06035-1

**Published:** 2018-09-03

**Authors:** Shula Chen, Yuqing Huang, Dennis Visser, Srinivasan Anand, Irina A. Buyanova, Weimin M. Chen

**Affiliations:** 1 Department of Physics, Chemistry and Biology, Linköping University, SE58183 Linköping, Sweden; 2 Department of Applied Physics, KTH Royal Institute of Technology, SE16440 Kista, Stockholm Sweden

## Abstract

Owing to their superior optical properties, semiconductor nanopillars/nanowires in one-dimensional (1D) geometry are building blocks for nano-photonics. They also hold potential for efficient polarized spin-light conversion in future spin nano-photonics. Unfortunately, spin generation in 1D systems so far remains inefficient at room temperature. Here we propose an approach that can significantly enhance the radiative efficiency of the electrons with the desired spin while suppressing that with the unwanted spin, which simultaneously ensures strong spin and light polarization. We demonstrate high optical polarization of 20%, inferring high electron spin polarization up to 60% at room temperature in a 1D system based on a GaNAs nanodisk-in-GaAs nanopillar structure, facilitated by spin-dependent recombination via merely 2–3 defects in each nanodisk. Our approach points to a promising direction for realization of an interface for efficient spin-photon quantum information transfer at room temperature—a key element for future spin-photonic applications.

## Abstract (web summary)

Room-temperature spin-generation in 1D systems like semiconductor nanopillars is typically inefficient. Here, the authors demonstrate an approach to achieve efficient spin polarization, even in the absence of a magnetic field, by selectively enhancing the radiative efficiency of one spin direction.

## Introduction

Semiconductor nanopillars (NPs)/nanowires (NWs) in the 1D geometry and their artificially designed lattices, like periodic arrays, represent a new class of metamaterials. They exhibit interesting optical properties superior to their flat-surface counterparts of semiconductor materials in the 3D and 2D geometry, such as enhanced light absorption and emission, waveguiding of light and light confinement thanks to a high refractive index contrast with respect to the surroundings (e.g., air), a large surface-to-volume ratio, etc. In the past decade, they have been extensively investigated as building blocks in nano-photonics, nano-photovoltaics, and nano-sensing^1,2^, including light emitting diodes (LEDs)^3–5^, photovoltaic (PV) cells^6–9^, photo-detectors^10–13^, nanolasers^14,15^, nonlinear optics^16–19^, and bio-sensors^20–22^, all on the nanoscale. NPs/NWs also function as natural axial Fabry-Perot (FP) and radial whispering-gallery (WG) mode cavities with tailorable optical modes^23,24^. The confinement of photonic modes and their propagation along the NP/NW axis, promising for guiding light on the nanoscale between different photonic components, offer great potential for applications in photonic integrated circuits. These superior optical properties have also placed NPs/NWs in a pole position to be ideal interfaces and building blocks bridging between spin- and photon-coded quantum information in future spin-photonic devices and integrated circuits.

A prerequisite for the success of such spin-photonic devices is efficient generation of spin-polarized carriers in NPs/NWs. Unfortunately, reports of spin generation in NPs/NWs remain sparse, in sharp contrast to numerous studies being carried out in semiconductors in the form of thin films^25–27^, quantum wells (QWs)^27–29^ and QW lasers^30–32^, and quantum dots (QDs)^33–35^. The earlier studies in NPs/NWs have focused on all electrical spin-FET and have largely been restricted to low temperatures in addition to requiring an external magnetic field^36–39^. To date, the highest electron spin polarization achieved in a 1D system at room temperature (RT) is 28.2% from a blue nanolaser based on GaN NWs in an applied magnetic field^40^. At present, generation of highly spin-polarized electrons at RT represents a major challenge in exploring the spin degree of freedom in NPs/NWs.

Moreover, a polarized spin-photon interface must exhibit both strong spin polarization and high radiative efficiency. These two requirements have been found to be difficult to meet simultaneously in a semiconductor system, as the conditions favoring spin polarization are generally unfavorable for radiative efficiency or vice versa (see below for details). Moreover, the maximum spin polarization achievable in a conventional approach is restricted to the initially injected spin polarization which is commonly rather low. This imposes a seemingly fundamental obstacle in realization of an efficient polarized spin-photon interface.

Here, we employ an approach that can effectively overcome the obstacle by dissociating the radiative efficiencies of the majority and minority spins, i.e., by significantly enhancing the radiative efficiency of the emitter with the desired spin while suppressing that with the unwanted spin, which simultaneously ensures strong electron spin polarization. We report on our achievement of electron spin polarization up to 60% at RT without a magnetic field, which is in fact much higher than the initially injected spin polarization, in a periodic array of dilute nitride GaNAs nanodisk (ND)-in-GaAs NP structure (referred to as GaNAs/GaAs DiP hereafter). We show that such a high electron spin polarization degree is facilitated by spin-dependent recombination (SDR) via, strikingly, merely 2–3 intrinsic point defects (i.e., gallium interstitials Ga_i_) in each GaNAs ND. Furthermore, we demonstrate that the NP array outperforms its planar counterpart in terms of both light absorption and detection efficiency thanks to the photonic crystal type of arrangement and the waveguide character of the NP array, which efficiently converts the electron spin polarization to polarized light in the fundamental HE_11_ cavity mode suitable for fiber-optic coupling. This work therefore demonstrates the potential of the proposed approach in the realization of RT-operational, polarized nanoscale spin and light emitters, e.g., the GaNAs/GaAs DiP structure, promising for practical applications in future spin-photonics and quantum communication network technology.

## Results

### GaNAs/GaAs DiP array design

**Fig. 1:**
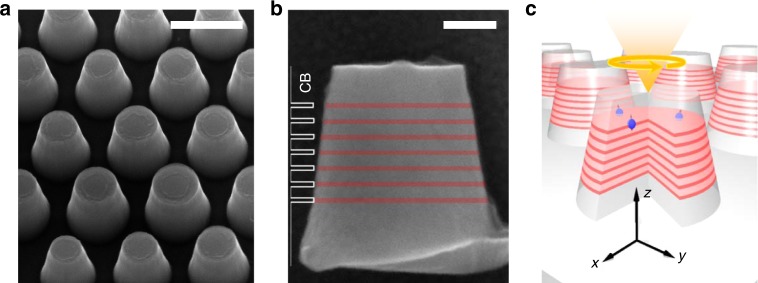
GaNAs/GaAs nanodisk in nanopillar array design. **a** Scanning electron microscopy (SEM) image of the arrayed GaNAs nanodisk-in-GaAs nanopillars (DiP). **b** Side-view SEM image of an individual DiP, where the seven GaNAs nanodisks are shown in red color. The energy profile of the conduction band (CB) edge along the nanopillar axis is indicated on the left side. The valence band edge is nearly unperturbed by N alloying or strain, and therefore its profile is approximately flat and not shown here. The scale bars in **a** and **b** denote 400 and 100 nm, respectively. **c** Schematic illustration of the experimental configuration

The GaNAs/GaAs DiP array was fabricated from a multiple quantum well (MQW) sample by a top-down approach employing nanosphere lithography (NSL) and inductively coupled plasma reactive ion etching (ICP-RIE) processes, see Methods section for details. The MQW sample contains seven GaNAs QWs, each with a thickness of 9 nm. The GaAs barrier between the neighboring QWs has a thickness of 20 nm. The N content varies over the range of 1.1–1.32% among the seven GaNAs QWs, see Supplementary Figure 1. Figure 1a shows a scanning electron microscopy (SEM) image of the as-etched DiP array, which exhibits a hexagonal lattice with an inter-pillar distance, *a*_0_, of ~460 nm as defined by the hexagonal close-packed nanosphere mask. Each NP features a tapered cylindrical shape with smooth side walls and a rather uniform size distribution, manifesting high quality of the nanofabrication. From the magnified SEM image on an individual NP shown in Fig. 1b, the average diameter, *d*, of the NPs is found to gradually increase from the top, *d*_top_ = 240 ± 10 nm, to the bottom, *d*_bottom_ = 360 ± 10 nm, over the height, *h*, of 370 ± 10 nm. The seven etched dilute nitride GaNAs NDs within each NP are highlighted in red, with the first ND at a distance of 70 nm from the top surface. The periodic modulation of the conduction band (CB) edge along the NP axis is depicted on the left side of Fig. 1b, where the CB energy is lower in the NDs due to incorporation of N^41^. The energy of the valence band (VB) edge of the NPs, on the other hand, is rather unperturbed^42^ (not shown here), as the incorporated N states predominantly interact with the CB and the alloy-induced strain in the NDs is relaxed owing to the DiP geometry (see Supplementary Note 1).

### Enhanced light absorption and extraction

**Fig. 2:**
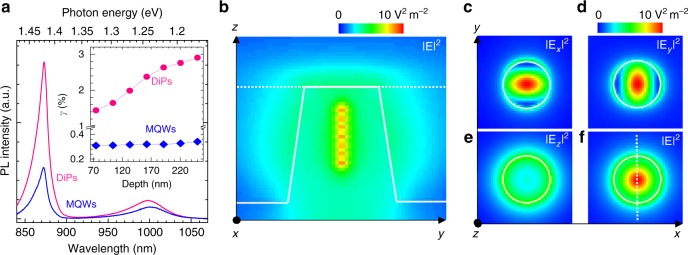
Enhanced light absorption and extraction at room temperature. **a** Photoluminescence (PL) spectra from the GaNAs nanodisk-in-GaAs nanopillars (DiPs) and the multiple quantum wells (MQWs) structure under unpolarized laser excitation at 800 nm. The inset shows the detection efficiency, *γ*, of both structures as a function of the dipole-emitter depth, i.e., the nanodisk (ND)/QW position from the top surface. **b** The side view of the finite difference time domain (FDTD) simulations of the squared electric-field amplitude (proportional to the light intensity) from the GaNAs NDs with distances of 75, 104, 133, 162, 191, 220, and 250 nm from the top surface. The simulation was taken at the middle cross-section of the DiP as marked by the dashed line in **f**. **c**–**e** The top views of the FDTD simulations of the ND light emission intensity with *x*, *y*, and *z* polarization components, respectively, together with that of the total PL intensity (**f**). The images are obtained in the plane indicated by the dashed line in **b**

To investigate the effect of the NP array on the light emission, we measured RT photoluminescence (PL) from the GaNAs/GaAs DiP under unpolarized laser excitation at 800 nm and compare it with the unetched reference MQWs. The results are shown in Fig. 2a. The PL spectra from both DiP and reference MQWs are dominated by two peaks, i.e., the shorter-wavelength one at 870 nm from the GaAs barrier and the broader one at the longer-wavelength of 1000 nm from the GaNAs NDs. Interestingly, the integrated PL intensities from GaNAs and GaAs of the DiP are stronger than that in the planar MQW structure by 1.55 and 3.04 times, respectively, under the same laser power *P*_exc_. Given that the etched DiP has a smaller emitting volume than the MQWs and it additionally suffers from enhanced non-radiative surface recombination associated with the unpassivated NP surface, the observed stronger PL from the DiP array clearly demonstrates a better light absorption and/or extraction efficiency than the planar MQWs. This finding is consistent with the PL results from other semiconductors with similar structures^43,44^. The measured PL intensity, *I*_PL_, is affected by multiple factors^45^,

1 $$I_{{\mathrm{PL}}} \propto P_{{\mathrm{exc}}}\alpha _{{\mathrm{in}}}V\eta \alpha _{{\mathrm{out}}}\beta,$$

where *α*_in_(*α*_out_) is the in (out)-coupling coefficient which determines the absorption (extraction) efficiency of light. The effective volume of the emitter is denoted by *V*, which is proportional to the material filling factor, *f*, i.e., *V*∝*f*. Here, *f* is 1 for the reference MQW sample and 0.38 for the DiP structure that is averaged over seven size-tapered GaNAs NDs. The radiative recombination efficiency (or the internal quantum efficiency) is denoted by *η*, and *β* represents the PL collection coefficient, i.e., the fraction of far-field emission that enters the numerical aperture (NA)-defined solid angle of a microscope objective. Based on the reflectivity measurements (see Supplementary Figure 2), we derive *α*_in_ = 0.94 for the DiP structure and *α*_in_ = 0.65 for the MQW sample, at the laser wavelength of 800 nm. From the temperature-dependent PL measurements (see Supplementary Figure 3), the GaNAs NDs in the DiP show a quantum efficiency of *η* = 0.01, which is lower than *η* = 0.03 of the MQWs due to surface recombination. We note that these values were obtained under unpolarized laser excitation, i.e., under the condition when the SDR effect is inactive. To gain quantitative insight into NP-tailored light output, we performed finite difference time domain (FDTD) calculations using Lumerical’s FDTD software. The PL radiation from the GaNAs NDs is modeled as unpolarized point-dipole emitters at 1000 nm, considering the band-to-band (BB) recombination involving both heavy-holes (HH) and light-holes (LH). The results are displayed in Fig. 2b for a single NP. The DiP array not only provides a vast density of nanostructured surface for efficient subwavelength light diffraction, but also it gives rise to a reduced contrast of effective refractive index with air, *n*_eff_, which leads to higher light transmission into free space^46,47^. More importantly, the shape of the DiP forms a free-standing waveguide which can couple the emission into the cavity mode. Simulations on the emission intensity across the NP cross-section given in Fig. 2c–f show that light is mostly confined at the center of the NPs, indicating a fundamental HE_11_-mode dominated PL output. All these factors can contribute to a higher PL detection efficiency *γ*, defined by *γ* = *α*_out_*β*, for the DiP than the planar MQWs. To confirm this, we calculated *γ* as a function of dipole emitter depth (or ND position) from the top surface for the two sample structures. As shown in the inset of Fig. 2a, *γ* of the DiP increases with increasing ND depth, reaching a maximum of 2.9% for the deepest ND. Its averaged *γ* of 2.3% is almost eight times higher than *γ* = 0.3 % for the MQWs. By applying all these derived parameters into eq. 1, the detected PL intensity from the GaNAs NDs in the DiP is calculated to be 1.4 times stronger than that from the MQWs, which is quite close to the value of 1.55 deduced from the PL results. The good agreement between the experiment and simulations verifies the optimized light management in the DiP structure with a promoted light-matter interaction and mode-profiled PL output. The obtained superior nano-photonic properties identify the GaNAs/GaAs DiP as an ideal emitter to couple with fiber-optics-based quantum networks or in photonic integrated circuits.

### Efficient RT spin generation enabled by few defects

**Fig. 3:**
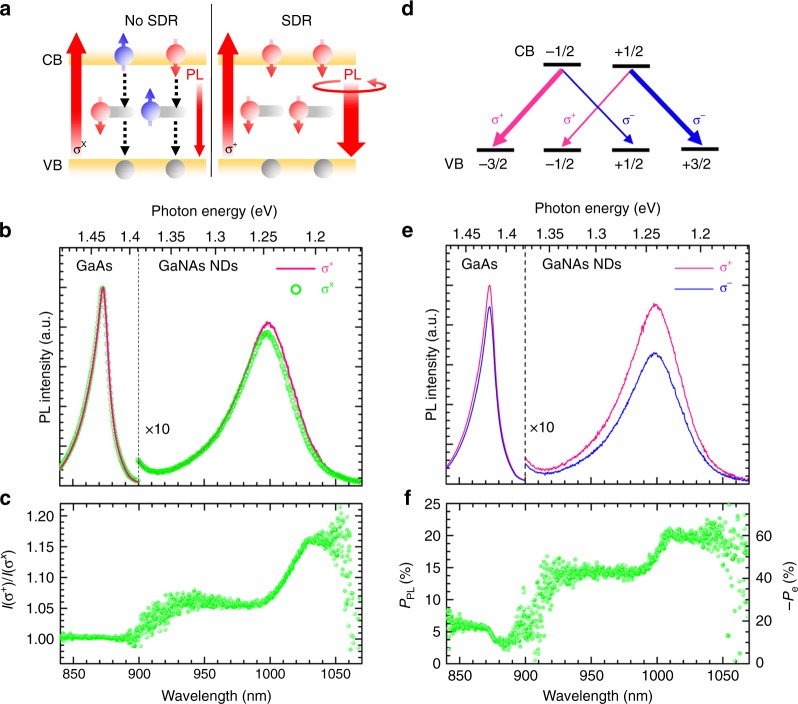
Efficient room temperature spin generation enabled by few defects. **a** Schematic illustration of the defect-driven spin generation via spin-dependent recombination (SDR) under σ^+^ excitation (the right panel), which shows stronger and co-polarized photoluminescence (PL) than that without the SDR effect under σ^x^ excitation (the left panel). The gray horizontal bars represent the defect level. **b** PL spectra excited by σ^+^ and σ^x^ laser light. **c** Spectral dependence of the *I*(σ^+^)/*I*(σ^X^) ratio. **d** Band diagram illustrating the polarized band-to-band transitions involving valence band (VB) heavy-hole (±3/2) and light-hole (±1/2) states. **e** σ^+^ and σ^−^ polarized PL spectra under the σ^+^ excitation. **f** Spectral dependence of the PL and conduction band (CB) electron spin polarizaion. In converting *P*_PL_–*P*_e_, the light depolarization factor arising from the finite solid angle of light detection by the microscope objective with the given numerical aperture (NA) has also been taken into account. Since, the PL emission from GaAs is at least partly contributed from the planar GaAs substrate, *P*_e_ in GaAs could be overestimated by a factor up to 1.5

Besides the advantage of enhanced light absorption and detection as established above, we shall now demonstrate efficient RT spin generation in the same GaNAs/GaAs DiP via the defect-mediated SDR processes. The Ga_i_ interstitial defects have been shown to be responsible for SDR in GaNAs from the earlier optically detected-magnetic-resonance (ODMR) study^27^. The principle of the spin generation via SDR is illustrated in Supplementary Figure 4 and described in more detail below, which relies on electron spin polarization of the ${\mathrm{Ga}}_{\mathrm{i}}^{2 + }$ defects with a single bound electron. If a preferential spin orientation of CB electrons is created by circular-polarized (σ^+^ or σ^−^) light, even only slightly, SDR will lead to a buildup of spin polarization of both CB and defect electrons during the optical pumping. For example, under σ^+^ light excitation along the NP axis as shown in Fig. 1c, a spin imbalance is generated for CB electrons with more spin-down than spin-up CB electrons as shown in Supplementary Figure 4, following the optical selection rule of the BB transition (Fig. 3d). This imbalance can be characterized by the ratio *n*_−_/*n*_+_ > 1 between the numbers of the spin-down and spin-up CB electrons, denoted by *n*_−_ and *n*_+_, respectively. Dictated by the Pauli exclusion principle, the capture of a CB electron by the ${\mathrm{Ga}}_{\mathrm{i}}^{2 + }$ defect is allowed only if the CB electron and the electron bound to ${\mathrm{Ga}}_{\mathrm{i}}^{2 + }$ have opposite spin orientations. As the probability of the spin-up (spin-down) ${\mathrm{Ga}}_{\mathrm{i}}^{2 + }$ defect in capturing a CB electron scales with the number of spin-down (spin-up) CB electrons, the spin-up ${\mathrm{Ga}}_{\mathrm{i}}^{2 + }$ defect should thus have a higher probability by a factor of *n*_−_/*n*_+_ in capturing a spin-down CB electron than the spin-down ${\mathrm{Ga}}_{\mathrm{i}}^{2 + }$ defect in capturing a spin-up CB electron, as illustrated in Supplementary Figure 4a. After the capture, one of the two spin-paired electrons at the ${\mathrm{Ga}}_{\mathrm{i}}^{1 + }$ defect will recombine with a spin-unpolarized VB hole, leaving behind with an equal probability either a spin-up or a spin-down electron at the defect. (Here, the VB hole spins are regarded as unpolarized in the SDR processes due to a stronger spin–orbit interaction and HH-LH coupling that randomize the initially polarized hole spins within picoseconds).^48,49^. As a result, after one round of the SDR process, more spin-up ${\mathrm{Ga}}_{\mathrm{i}}^{2 + }$ defects are converted to the spin-down ${\mathrm{Ga}}_{\mathrm{i}}^{2 + }$ defect than the other way around, with a factor of *n*_−_/*n*_+_ between their conversion rates. This leads to an imbalance between spin-down and spin-up ${\mathrm{Ga}}_{\mathrm{i}}^{2 + }$ defects, as illustrated in Supplementary Figure 4b, which can be characterized by their ratio *N*_−_/*N*_+_ > 1. With each round of such capture and recombination, more and more spin-up ${\mathrm{Ga}}_{\mathrm{i}}^{2 + }$ defects will be converted to the spin-down ${\mathrm{Ga}}_{\mathrm{i}}^{2 + }$ defects. At the same time, an increasingly larger ratio *N*_−_/*N*_+_ will in turn preferably deplete the spin-up (minority spin) CB electrons, i.e., a spin filtering effect, thereby further increasing the ratio *n*_−_/*n*_+_ and thus the CB electron spin polarization. Eventually this positive feedback process between *n*_−_/*n*_+_ and *N*_−_/*N*_+_ will drive both the ${\mathrm{Ga}}_{\mathrm{i}}^{2 + }$ defect and CB electron spins towards the majority-spin (i.e., spin-down) orientation via the so-called dynamic spin polarization (DSP) process^50^. As shown in the right panel of Fig. 3a, the resulting spin blockade effect prevents further capture and depletion of CB electrons via the defects, thereby giving rise to a stronger and highly circular-polarized BB emission. During the time when the defect electron spin is preserved, any minority-spin CB electrons generated by either poor optical spin generation by light or due to spin relaxation will immediately be depleted by the SDR processes via the defects as illustrated in Supplementary Figure 4c. This ensures that spin polarization of CB electrons can be maintained at a substantially higher degree than the initial value created by optical pumping without DSP. This is in sharp contrast to the case without SDR and DSP under linear-polarized (σ^X^) laser excitation, see the left panel of Fig. 3a, when both spin-up and spin-down electrons are equally generated in CB, followed by their capture with an equal probability by the spin-unpolarized Ga_i_ defects and recombination with VB holes. No spin polarization of either the defect electrons or CB electrons is expected during the capture and recombination processes, except that this fast non-radiative process equally depletes free carriers of both spin orientations resulting in weak and unpolarized PL emission.

The most crucial condition for the success of the SDR and DSP processes in efficient spin generation is *τ*_sc_ > *τ*_s_ > *τ*_nr_. Here, a much longer spin lifetime of the defect electron, denoted by *τ*_sc_, ensures that the ${\mathrm{Ga}}_{\mathrm{i}}^{2 + }$ defects remain spin polarized during the time when CB electrons undergo spin relaxation. Whenever that happens, the defects will immediately filter out these spin-flipped CB electrons as long as their capture time by the defects (*τ*_nr_) is shorter than the CB electron spin relaxation time *τ*_s_. As *τ*_nr_ is typically in the order of ps, the spin filtering and spin amplification can be accomplished within a very short time. The efficiency of the SDR and DSP processes can also be accelerated by increasing circularly polarized light intensity because it is accompanied by an increasing probability of the ${\mathrm{Ga}}_{\mathrm{i}}^{2 + }$ defects in capturing CB electrons and thus an increasing number of the SDR cycles within the same period of time.

The boost in the PL intensity due to the spin blockade effect can be characterized by the ratio of *I*(σ^+^ or σ^−^)/*I*(σ^X^), where *I*(σ^+/−/X^) represents the PL intensity under right-circular/left-circular/linear-polarized laser excitation, respectively. Figure 3b shows representative PL spectra excited by the 800 nm laser line at *P*_exc_ of 11.7 mW, where the SDR process in the GaNAs NDs is clearly evident from an apparently stronger emission under σ^+^ than σ^X^ excitation. This can be further corroborated with an unchanged PL signal from GaAs, where no SDR occurs. The corresponding *I*(σ^+^)/*I*(σ^X^) ratio in Fig. 3c clearly exhibits a variation from a value of ~1.06 to ~1.15 with decreasing emission energy. The spectral variation of the *I*(σ^+^)/*I*(σ^X^) ratio suggests the coexistence of SDR processes with different efficiencies. Indeed, our secondary ion mass spectroscopy (SIMS) measurements on the reference MQWs reveal a *N* fluctuation from [*N*_min_] = 1.1% to [*N*_max_] = 1.32% among the seven QWs (see Supplementary Figure 1), corresponding to a bandgap energy shift from 1.252 to 1.228 eV which is comparable with the spectral width of the broad GaNAs PL. Since a larger *N* content not only reduces the bandgap energy but also facilitates the formation of Ga_i_^27,51^, and thus SDR efficiency, a higher *I*(σ^+^)/*I*(σ^X^) ratio is expected at the lower emission energy that is in excellent agreement with our experimental finding. The lowest and highest values of the *I*(σ^+^)/*I*(σ^X^) ratio are thus derived from the NDs with [*N*_min_] and [*N*_max_], respectively.

To directly determine spin polarization of CB electrons in the GaNAs NDs, we monitored the circular-polarized PL components under circularly polarized optical pumping (e.g., σ^+^). The ND emission is strongly co-polarized with the excitation light as shown in Fig. 3e. The derived PL circular polarization degree *P*_PL_, defined by *P*_PL_ = (*I*^+^−*I*^−^)/(*I*^+^+*I*^−^) where *I*^+/−^ denotes the σ^+/−^-polarized PL intensity, is shown in Fig. 3f. It exhibits a spectral variation similar to the *I*(σ^+^)/*I*(σ^X^) ratio, with *P*_PL_ = 14 and 20% from the NDs with [*N*_min_] and [*N*_max_], respectively. It should be noted that due to the degeneracy of HH and LH VB states in the strain-relaxed NDs, see Supplementary Note 1 and Supplementary Figure 5–6, the detected *P*_PL_ comprises contributions from both HH and LH subbands of opposite helicity. Based on the well-known 3-to-1 ratio between the oscillator strengths of the BB optical transitions involving the HH and LH states^48,52^, as illustrated in Fig. 3d, the spin polarization of CB electrons is twice of *P*_PL_, i.e., *P*_e_ = −2*P*_PL_. By further taking into account the light depolarization factor arising from the finite solid angle of light detection by the microscope objective with the given NA (see Supplementary Figure 7 and Supplementary Note 2), *P*_e_ generated in the GaNAs NDs with [*N*_min_] = 1.1% to [*N*_max_] = 1.32% can be determined to be 43 and 60%, respectively. These values are estimated based on the results from Supplementary Figure 7 that 100% electron spin polarization should generate only 33% light circular polarization under our experimental conditions and assuming full LH-HH degeneracy.

**Fig. 4:**
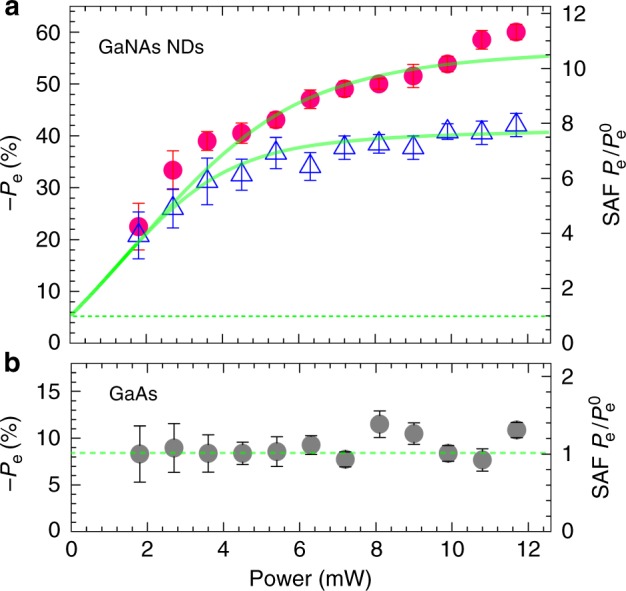
Room temperature spin amplification enabled by few defects. **a** Power-dependent *P*_e_ and spin amplification factor ${\mathrm{SAF}} = P_{\mathrm{e}}{\mathrm{/}}P_{\mathrm{e}}^0$ from the GaNAs nanodisks (NDs) with the [*N*_min_] (the open triangles) and [*N*_max_] (the filled circles). The solid symbols are experimental data, and the solid lines are the fitting curves obtained by the rate equation analysis. **b** For comparison, power-dependent *P*_e_ from GaAs is also shown by the gray symbols. The vertical bars indicate the experimental error bars which are derived from the statistics of five measurements. The dashed horizontal line marks $P_{\mathrm{e}}^0$

To evaluate to what extent the SDR effect via the defects have contributed to the spin generation in the DiP, we carried out a detailed study of excitation power-dependent *P*_e_ and *I*(σ^+^)/*I*(σ^X^) ratio for the NDs with [*N*_min_] and [*N*_max_]. A summary of the results is shown in Fig. 4. For both *N* compositions, *P*_e_ increases with increasing *P*_exc_ owing to accelerated dynamic processes of SDR. This can also be corroborated by a constant *P*_e_ of ~8% for the GaAs emission, where the SDR effect is absent. From the best fits of a nonlinear coupled rate equation analysis to the power-dependent *P*_e_ profiles, see Supplementary Note 3 for details, the concentration of the Ga_i_ defects can be estimated to be ~2.6 × 10^15^ cm^−3^ and 4.1 × 10^15^ cm^−3^ in the NDs with [*N*_min_] and [*N*_max_], respectively. This approximately corresponds to 2 defects in each ND with [*N*_min_] and 3 defects in each ND with [*N*_max_]. It is remarkable that such few defects have led to strong *P*_e_ up to 60% at RT, demonstrating the extremely high efficiency and robustness of the Ga_i_-mediated spin generation processes.

The rate equation analysis also shows that the spin polarization at very low *P*_exc_ before the optically pumped dynamic spin polarization process of the defect electrons takes place, denoted by $P_{\mathrm{e}}^0$, is merely 5%. This value reflects the CB electron polarization without the SDR effect. The low $P_{\mathrm{e}}^0( { = {\textstyle{{P_{\mathrm{e}}^{\mathrm{i}}} \over {1 + \tau /\tau _{\mathrm{s}}}}}} )$ in the GaNAs NDs, much lower than the maximum allowed 50% upon optical pumping of the GaAs barrier, originates from two parts. The first part stems from spin loss of the light emitter itself, by a factor of ${\textstyle{1 \over {1 + \tau /\tau _{\mathrm{s}}}}}$ (about 0.9 in our case), due to CB electron spin relaxation within its lifetime. The second contribution, often the more important one, is related to a low value of $P_{\mathrm{e}}^{\mathrm{i}}$ (about 5.6% in our case), which reflects significant spin loss during energy relaxation of hot electrons/excitons within the GaAs barriers before spin injection to the GaNAs NDs, spin transport across the GaAs/GaNAs interfaces, and energy relaxation with the NDs after spin injection. In fact, it is such severe spin loss that has prevented GaAs, or any other non-magnetic semiconductor without SDR, from efficient spin generation at RT. When the SDR effect is activated as *P*_exc_ increases, *P*_e_ increases and eventually approaches a saturation value of 60% (43%) for the NDs with [*N*_max_] ([*N*_min_]) due to the saturated SDR process. The observed defect-enabled spin gain can be characterized by a spin amplification factor (SAF), i.e., ${\mathrm{SAF}} = P_{\mathrm{e}}{\mathrm{/}}P_{\mathrm{e}}^0$, yielding a maximum SAF of 11 (7.6) for the NDs with [*N*_max_] ([*N*_min_]). These findings further consolidate the effectiveness of the Ga_i_ defects for spin amplification in the GaNAs NDs, which could effectively overcome the severe spin loss in the way no any other approach can.

### Spin polarization versus internal quantum efficiency

An efficient polarized spin-photon interface must fulfill two stringent requirements: strong electron spin polarization and efficient light emitting. In a most commonly occurring case of a semiconductor without the defect-enabled spin amplification effect, these two requirements favor contradicting conditions. This is because the electron spin polarization ${P}_{\mathrm{e}}^0$ can be described in its simplest form by the equation^52^

2 $$P_{\mathrm{e}}^0 = \frac{{P_{\mathrm{e}}^{\mathrm{i}}}}{{1 + \tau /\tau _{\mathrm{s}}}}.$$

**Fig. 5:**
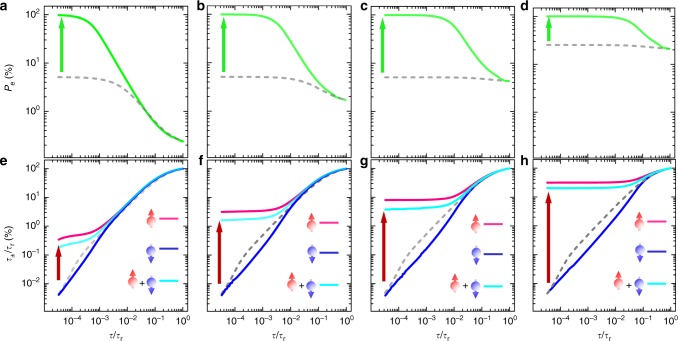
Spin polarization versus internal quantum efficiency. **a**–**d** Conduction band electron spin polarization *P*_e_. **e**–**h** Radiative recombination efficiency of the majority-spin (spin-up) and minority-spin (spin-down), *η*_±_ = *τ*_±_/*τ*_r_, as well as the total radiative recombination efficiency. They are plotted as a function of *τ*/*τ*_r_, calculated from the rate equation analysis using the following parameters: **a**, **e** *τ*_sc_ = 2*τ*_s_, *τ*_r_ = 10*τ*_sc_, $P_{\mathrm{e}}^0 = 5{\mathrm{\% }}$; **b**, **f** *τ*_sc_ = 2*τ*_s_, *τ*_r_ = *τ*_sc_, $P_{\mathrm{e}}^0 = 5{\mathrm{\% }}$; **c**, **g** *τ*_sc_ = 2*τ*_s_, *τ*_r_ = 0.1*τ*_sc_, $P_{\mathrm{e}}^0 = 5{\mathrm{\% }}$; **d**,**h**, *τ*_sc_ = 2τ_s_, *τ*_r_ = 0.1*τ*_sc_, $P_{\mathrm{e}}^0 = 25{\mathrm{\% }}$. *τ*_r_ is fixed at 3000 ps in all cases, and ${\textstyle{1 \over \tau }} = {\textstyle{1 \over {\tau _{\mathrm{r}}}}} + \gamma _{\mathrm{e}}N_{\mathrm{c}}$. The simulations corresponding to the cases with (without) the defect-mediated SDR are displayed by the colored solid lines (the gray dashed lines). The green and red vertical arrows indicate the enhancements of electron spin polarization and radiative recombination efficiency induced by the few-defect-mediated SDR effect, respectively

Here, *τ* denotes the total lifetime of the electrons regardless of their spin orientations, which is contributed from both radiative and non-radiative lifetime (i.e., *τ*_r_ and *τ*_nr_) following the relationship ${\textstyle{1 \over \tau }} = {\textstyle{1 \over {\tau _{\mathrm{r}}}}} + {\textstyle{1 \over {\tau _{{\mathrm{nr}}}}}}$. (${\textstyle{1 \over {\tau _{{\mathrm{nr}}}}}} = \gamma _{\mathrm{e}}N_{\mathrm{c}}$, where *N*_c_ is the defect concentration responsible for the non-radiative recombination and γ_e_ is the trapping coefficient of CB electrons by the defect.) From eq. 2, it is apparent that $P_{\mathrm{e}}^0$ is restrained over the range of ${\textstyle{{P_{\mathrm{e}}^{\mathrm{i}}} \over {1 + \tau /\tau _{\mathrm{s}}}}} \le P_{\mathrm{e}}^0 \le P_{\mathrm{e}}^{\mathrm{i}}$. Achieving the maximum radiative recombination efficiency *η* = *τ*/*τ*_r_ = 100% is accompanied by the minimum electron spin polarization $P_{\mathrm{e}}^0 = {\textstyle{{P_{\mathrm{e}}^{\mathrm{i}}} \over {1 + \tau _{\mathrm{r}}/\tau _{\mathrm{s}}}}}$. As *τ*_r_ is usually found to be comparable or longer than *τ*_s_ in semiconductor materials and nanostructures, the maximum *η* is obtained at the expense of severe spin loss. To approach the maximum value of $P_{\mathrm{e}}^0 \approx P_{\mathrm{e}}^{\mathrm{i}}$, on the other hand, *τ* needs to be much shorter than *τ*_s_. This requires introduction of non-radiative recombination such that *τ* ≈ *τ*_nr_ ≪ *τ*_s_^53^, which simultaneously minimizes *η*, as *η* = *τ*/*τ*_r_≪1 under the same condition. Such an anti-correlation between *P*_e_ and *η* can be clearly visualized from the simulations shown by the gray dashed lines in Fig. 5.

This seemingly fundamental obstacle in simultaneously achieving both strong *P*_e_ and high *η* can be effectively overcome by the defect-mediated SDR effect presented in this work. The key to the success here is a strong spin polarization degree of the defect electrons such that the non-radiative recombination times of the spin-up and spin-down CB electrons, namely, $\tau _{{\mathrm{nr}}}^ \pm = {\textstyle{1 \over {\gamma _{\mathrm{e}}N_ \mp }}}$, becomes markedly different. Here, *N*_±_ represents the concentration of the Ga_i_ defects in the paramagnetic charge state occupied by a single electron with the subscript ‘±’ denoting the spin-up and spin-down orientation of electrons. In the extreme case when the defect electrons are completely spin polarized, say *N*_+_ ≈ *N*_c_ and *N*_−_ ≈ 0, non-radiative recombination of the CB electrons with the majority-spin (i.e., spin-up) diminishes since $\tau _{{\mathrm{nr}}}^ + = {\textstyle{1 \over {\gamma _{\mathrm{e}}N_ - }}} \to \infty$ yielding *η*_+_→100%. This is in sharp contrast to the CB electrons with the minority-spin (i.e., spin-down) that suffer strong non-radiative recombination in the same way as the case without the SDR effect described above, because $\tau _{{\mathrm{nr}}}^ - = {\textstyle{1 \over {\gamma _{\mathrm{e}}N_ + }}}$. This strong deviation in non-radiative recombination via the defects between the majority-spin and minority-spin CB electrons in turn drives CB electrons toward amplified spin polarization ($P_{\mathrm{e}} > P_{\mathrm{e}}^{\mathrm{i}}$) well exceeding the maximum limit of $P_{\mathrm{e}}^0 \approx P_{\mathrm{e}}^{\mathrm{i}}$ imposed in the absence of the SDR effect. For a quantitative analysis, we performed detailed simulations of *P*_e_ and *η*_±_ = *τ*_±_/*τ*_r_ as a function of *τ*/*τ*_r_ with the aid of the coupled rate equations given in Supplementary Note 3. The results are shown in Fig. 5, which demonstrate that, over the regime when the SDR is highly active, both *P*_e_ and *η* (for the desired spins) can be significantly enhanced by orders of magnitude from that without the SDR effect. We should note that, as the minority-spin CB electrons are depleted by the defect-mediated non-radiative recombination processes, the total quantum efficiency *η*_total_, including PL yield from both majority-spin and minority-spin CB electrons, should lie between the quantum efficiency values of the two. The *η*_total_ values calculated based on the rate equations are shown as a function of *τ*/*τ*_r_ in Fig. 5, together with the values of the majority-spin and minority-spin quantum efficiency. The simulations confirm the significant enhancement of not only the majority-spin quantum efficiency but also total PL quantum yield by activating the SDR effect. The upper bound of *η*_total_ achievable by the defect-mediated SDR effect is determined by the number of minority-spin CB electrons needed to be depleted via the non-radiative processes. Theoretically, it can approach 50% when $P_{\mathrm{e}}^{\mathrm{i}}$ is close to zero and 100% in the other extreme case of $P_{\mathrm{e}}^{\mathrm{i}}$ close to 100%.

It is clear from the simulations shown in Fig. 5 that the exact extent of the enhancement in both *P*_e_ and *η* by the SDR effect critically depends on the defect concentration (thus *τ*), CB electron spin relaxation time *τ*_s_ and the initial spin polarization $P_{\mathrm{e}}^{\mathrm{i}}$. This provides a guideline for further improvements by increasing the SDR defect concentration and by suppressing CB electron spin relaxation through, e.g., quantum confinement. The simulations also show the potential of our approach to simultaneously fulfill both requirements for strong electron spin polarization with *P*_e_ approaching 100% and high radiative recombination efficiency in the studied DiP structure. Future effort is also required to improve polarized spin-light conversion by removing the HH-LH degeneracy–the source of partial cancellation (by 1/2) in emitting light polarization, through strain engineering and/or quantum confinement such that the BB transition at RT only involves the HH states.

We note possible alternative approaches to reduce the radiative lifetime by exploring the Purcell effect and spin lasers^54–57^. Indeed, these alternative approaches in principle have the potential to increase both *P*_e_ and *η*. However, as the Purcell effect is not spin selective and therefore is incapable of spin amplification, it can only suppress spin loss of the emitter by reducing *τ*/*τ*_s_. As a result, *P*_e_ is still fundamentally limited by the initial spin polarization of the injected electrons $P_{\mathrm{e}}^{\mathrm{i}}$, since $P_{\mathrm{e}}^0 = {\textstyle{{P_{\mathrm{e}}^{\mathrm{i}}} \over {1 + \tau /\tau _{\mathrm{s}}}}} \le P_{\mathrm{e}}^{\mathrm{i}}$ no matter how small value *τ*/*τ*_s_ is. Furthermore, to achieve the Purcell effect, either metal-based nanoplasmonics or dielectric Fabry-Perot/whispering-gallery cavities are required to reduce the spontaneous radiative lifetime by increasing the light-emitter interaction. It is commonly known that metal-based nanoplasmonics introduces additional Ohmic loss and could in addition cause severe depolarization of the circularly polarized emitting light from a 1D structure, which are undesirable for a spin-photon interface. Spin-lasers are attractive as they are capable of spin amplification under the injection condition when the lasing threshold for the majority spin is reached whereas it is not for the minority spin. The main drawback is that they involve more complex nanofabrication processes and require a high injection level (thus high power consumption). More critical is perhaps the requirement for well-separated injection levels at which the majority-spin and minority-spin start lasing^57^, which demands a high $P_{\mathrm{e}}^0$ before lasing, i.e., before the spin amplification takes place. For example, $P_{\mathrm{e}}^0$ = 20% in the spin laser based on GaAs/AlGaAs MQWs reported in ref.^57^. Considering CB electron spin polarization is typically rather weak in a common semiconductor at RT without spin amplification, e.g., $P_{\mathrm{e}}^0$ = 5% in our case of the GaNAs/GaAs DiPs, applications of spin lasers in material systems with a week $P_{\mathrm{e}}^0$, where spin amplification is most needed, could be rather restricted. In our work, we show both experimentally and theoretically that the SDR-mediated spin amplification process remains highly effective even with $P_{\mathrm{e}}^0$ = 5% or even lower. The most challenging task is to identify a suitable spin-filtering defect and to controllably incorporate them in a host lattice. In GaNAs, the spin-filtering Ga_i_ defects have been identified by the optically detected-magnetic resonance technique^27^ and their concentrations can be controlled by e.g., N composition, growth temperature and post-growth thermal annealing^58^.

## Discussion

In conclusion, we have proposed and realized an efficient novel polarized spin-light emitter at RT based on a high-quality periodic array of the GaNAs/GaAs DiP. We observed a high PL circular polarization degree of 20%, which corresponds to record-efficient spin generation of CB electrons in a semiconductor materials system with a 1D geometry, up to 60% at RT without an applied magnetic field. Strikingly, such achievement is enabled by the SDR effect via merely 2–3 defects in each ND. The functionality of the spin amplification with a spin gain up to 11 times at RT provided by such few defects is unique and could possibly be found indispensable to achieve nearly 100% electron spin polarization, which no any other approach is capable of. We showed that our approach holds the potential to simultaneously fulfill the requirements for both strong electron spin polarization and high internal quantum efficiency, thereby overcoming a seemingly fundamental limit imposed on semiconductors in this respect. Combining with an optimized light in- and out-coupling strength and the waveguide-tailored spin output for fiber-optic interconnection, the nano-photonic structure of the GaNAs/GaAs DiP is promising for a highly sensitive and efficient nano-sized interface for spin-to-photon quantum information transfer at RT, paving the way for practical applications in future nanoscale spin-photonics and quantum communication networks.

The demonstrated defect-enabled spin generation and polarized spin-photon conversion also have the following added advantages: (i) the defect-mediated SDR and the resulting spin amplification processes are thermally activated, which warrants efficient and fast spin generation and manipulation at RT or even higher temperatures; (ii) the giant bandgap bowing effect accompanying dilute-alloying of GaAs with nitrogen in the GaNAs/GaAs DiP significantly shifts the emission wavelength toward the near infrared spectral range suitable for fiber-optic communications, while keeping the oscillator strength nearly unchanged as a bright emitter; (iii) the heterostructures embedded in the DiP structures provide an additional degree of freedom in bandgap engineering and strain engineering for optimal device performance; (iv) no magnetic fields required, thus averting design complexities that are often associated with incorporating local magnetic fields into device architectures; (v) freedom and ease in selecting and switching spin direction by properly orienting defect electron spins; (vi) availability of a rather mature technology base of GaAs, i.e., the parent material of GaNAs, which has widely been used today for high-frequency and optoelectronic devices.

## Methods

### Fabrication of the GaNAs/GaAs DiP array

We employed a cost-effective and mass-scalable top-down method, involving nanosphere lithography and inductively coupled plasma reactive ion etching processes^59^, to fabricate the dilute nitride GaNAs/GaAs DiP array. A monolayer of SiO_2_ nanospheres with a diameter of ~460 nm was dispersed by spin-coating onto a GaNAs/GaAs MQW sample, forming a hexagonal close-packed arrangement. The SiO_2_ nanosphere size was reduced (on-site) to ~300–350 nm by subsequent fluorine-based RIE for 8 min. After that ICP-RIE using Cl_2_/H_2_/CH_4_ chemistry was performed under optimized conditions for 2.5 min to etch the MQWs into a NP array. Finally, the top residual SiO_2_ NS mask was removed by HF etching.

### Experimental techniques

The PL measurements were performed on a Horiba micro-PL (µ-PL) setup. A continuous-wave (CW) Ti: Sapphire laser was used as an excitation source, with output at 800 nm to excite carriers across the GaAs bandgap. For optical orientation of CB electron spins, a set of linear polarizer and quarter-wave plate were used to create σ^+^ or σ^−^ excitation, which could generate a maximum *P*_e_ of 50% in GaAs according to the selection rule of the BB optical transitions involving the VB HH and LH states. A microscope objective, ×50/NA0.5, was used to focus the excitation beam along the NP axis (*z*-axis in Fig. 1c) to a spot size of ~4 µm^2^, by which ~20 NPs were excited. At the highest excitation power of 12 mW used in our experiments, the electron generation rate in each ND under the above and below GaAs excitation is estimated to be ~2 × 10^12^ s^−1^ (~2 ps^−1^) and ~1 × 10^12^ s^−1^ (~1 ps^−1^), respectively. The resulting PL signal from the GaNAs DiP array was dispersed by a single-grating monochromator and detected by a CCD camera. The circular-polarized emission components were resolved by a second set of linear polarizer and quarter-wave plate.

## Electronic supplementary material

Supplementary Information

## Data Availability

All relevant data are available from the authors upon request.
