# Supplementary material for: Room-temperature polarized spin-photon interface based on a semiconductor nanodisk-in-nanopillar structure driven by few defects
Source: Nat Commun. 2018 Sep 3;9:3575. doi: 10.1038/s41467-018-06035-1 (PMC6120900; doi:10.1038/s41467-018-06035-1)
Supplement: Supplementary file 1 — Supplementary Information [file 41467_2018_6035_MOESM1_ESM.pdf]

Supplementary Information for

Room-temperature polarized spin-photon interface based on a semiconductor nanodisk-in-nanopillar structure driven by few defects

Shula Chen,[1,] Yuqing Huang,[1] Dennis Visser,[2] Srinivasan Anand,[2] Irina A. Buyanova,[1] & Weimin M. Chen,[1,*]*

[1] Department of Physics, Chemistry and Biology, Linköping University, SE58183 Linköping, Sweden

[2] Department of Applied Physics, KTH Royal Institute of Technology, SE16440 Kista, Stockholm, Sweden

** Correspondence and requests for materials should be addressed to:*

W.M.C. (email: wmc@ifm.liu.se), S.C. (email: shuch@ifm.liu.se)

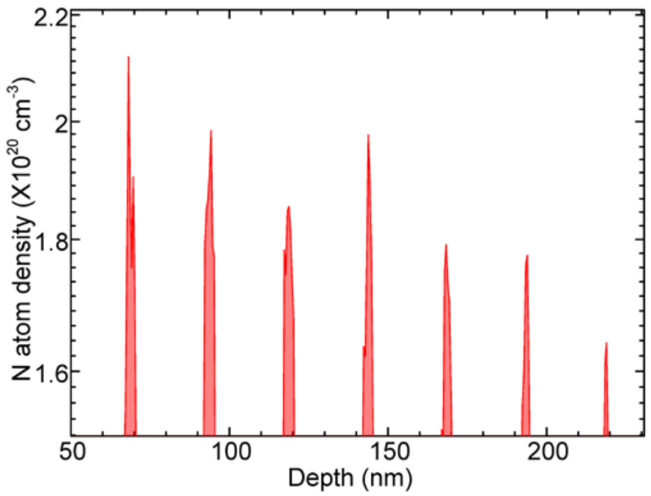

Supplementary Figure 1. **Secondary ion mass spectroscopy from the reference multiple quantum wells.** N composition varies from [N_{min}]=1.1% to [N_{max}]=1.32% among the seven quantum wells (QWs).

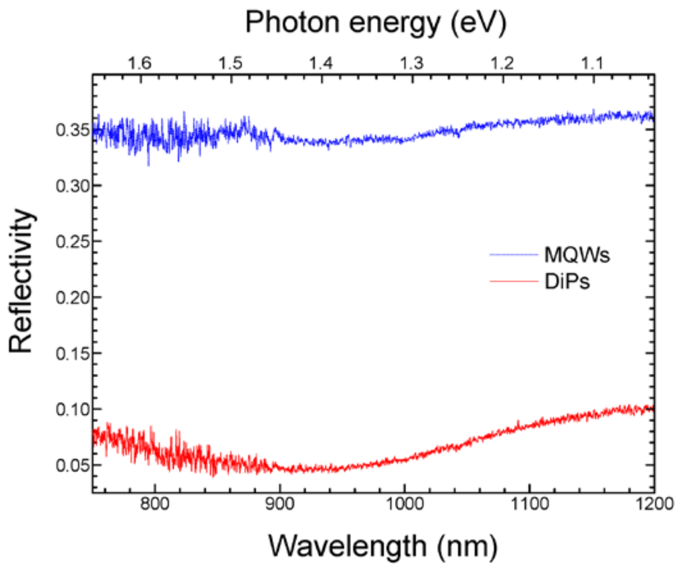

Supplementary Figure 2. Reflectivity measurements. Results from the reflectivity measurements from the multiple QWs (MQWs) and the GaNAs nanodisks-in-GaAs nanopillars (DiPs). The DiP array exhibits a much lower reflectance than the MQW structure owing to its periodically nanostructured surface. At the laser excitation wavelength of 800 nm, the MQW and DiP structures have a reflectance of ~ 0.35 and ~ 0.06, which corresponds to an in-coupling coefficient, α_{in}, of ~ 0.65 and ~ 0.94, respectively.

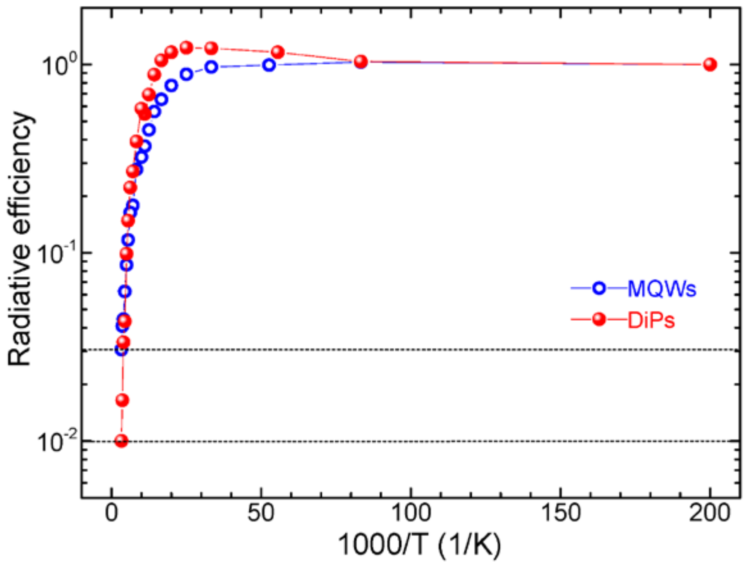

Supplementary Figure 3. Temperature-dependent photoluminescence. Temperature-dependent photoluminescence intensity of the GaNAs emission from the MQW and DiP structures. The horizontal dotted lines indicate the RT values of radiative recombination efficiency from the two structures. The data were taken under unpolarized laser excitation, i.e. under the condition when the spin-dependent recombination (SDR) effect is inactive.

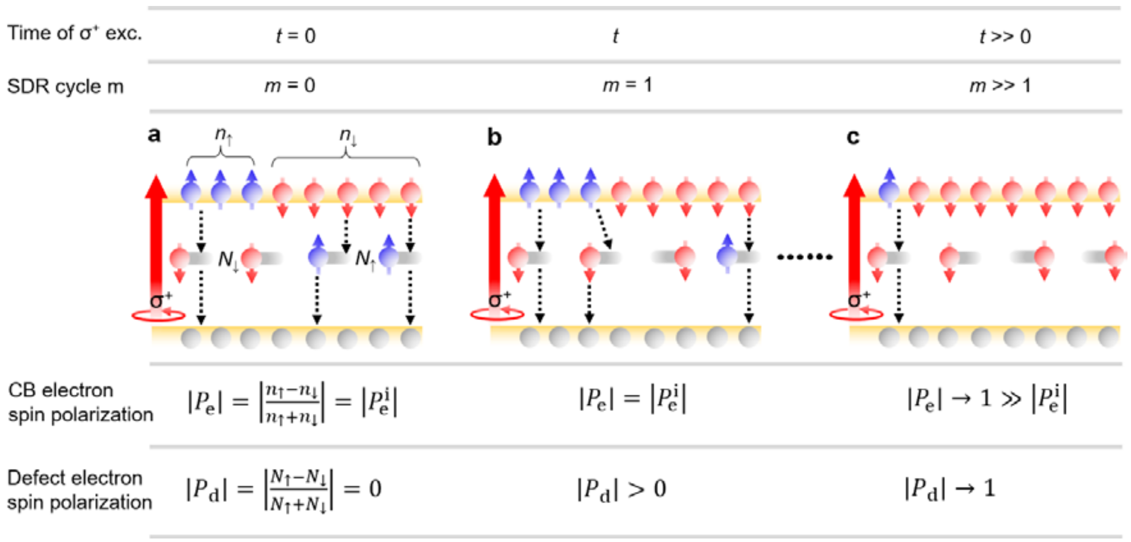

Supplementary Figure 4. Physical principle of the defect-mediated spin-dependent recombination and spin amplification. Schematic illustration of the physical principle of the defect-mediated SDR and spin amplification. σ^+ denotes right circular-polarized excitation. t and m represent the time length of optical spin orientation and corresponding rounds of SDR. $n_{\uparrow(\downarrow)}$ and $N_{\uparrow(\downarrow)}$ are the spin-up (spin-down) electron density in conduction band (CB) and defect states, respectively. P_e and P_e^i represent CB electron spin polarization and its initial value, while the spin polarization at defects is labelled as P_d.

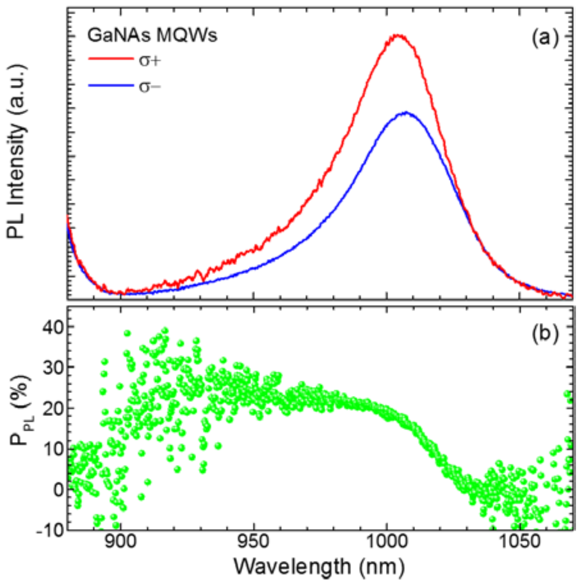

Supplementary Figure 5. **Photoluminescence polarization from the GaNAs multiple quantum wells.** σ^+ and σ^- polarized photoluminescence (PL) spectra (upper panel) and spectral dependence of the PL polarization (lower panel) obtained at RT from the reference sample of the GaNAs MQWs under the σ^+ excitation.

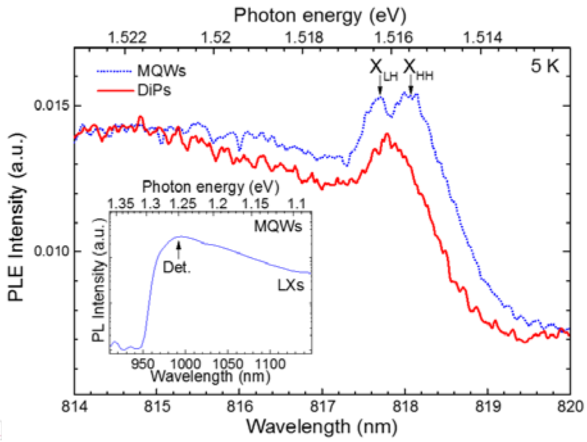

Supplementary Figure 6. Photoluminescence excitation. PL excitation (PLE) spectra measured at 5 K from both GaNAs MQW and DiP structures. The inset shows a typical localized exciton (LX) emission from the MQWs, where the detection energy in PLE is indicated by the arrow. By exciting above the GaAs bandgap, the detected LE emission in GaNAs mainly originates from injection of photo-generated carriers/excitons from GaAs within the diffusion range close to the GaAs/GaNAs interfaces. Due to the lattice mismatch between GaAs and GaNAs, a strain field could be expected to be present near the interfaces that could remove the HH-LH degeneracy in GaAs. The PLE spectrum from the MQWs clearly exhibits a doublet feature at the GaAs free exciton resonance, corresponding to the heavy-hole (HH) exciton (X_{HH}) and the light-hole (LH) exciton (X_{LH}) with the former lying at the lower energy due to a compressive in-plane strain the GaAs barrier experiences near the interfaces with GaNAs. In contrast, the PLE spectrum from the DiP structure shows only one peak, which is indicative of X_{HH} and X_{LH} degeneracy and, hence, strain relaxation in the DiP structure.

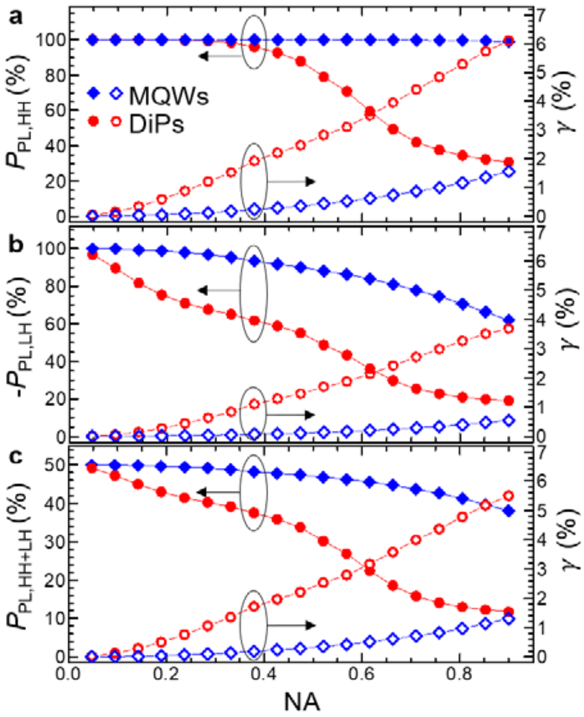

Supplementary Figure 7. Simulations of photoluminescence polarization and detection efficiency. Calculated PL circular polarization degree, P_{PL}, and detection efficiency, γ, of the DiP and MQW structures as a function of numerical aperture, NA. The calculations consider the PL transition involving (a) the HH VB state, (b) the LH VB state, and (c) degenerate HH-LH VB states.

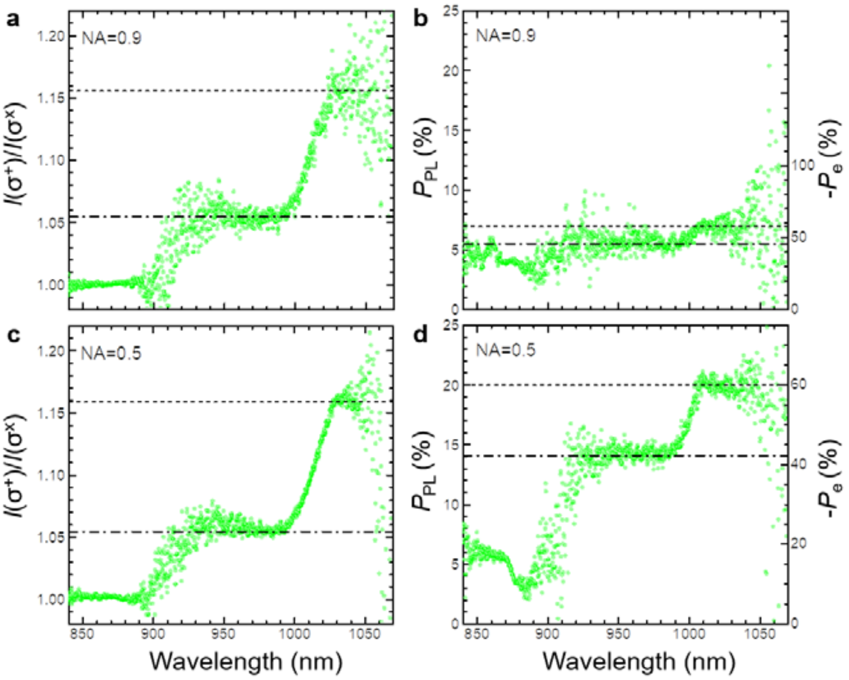

Supplementary Figure 8. $I(\sigma^+)/I(\sigma^X)$ ratio and photoluminescence polarization versus numerical aperture. Spectral dependence of the $I(\sigma^+)/I(\sigma^X)$ ratio (a) and PL polarization (b) obtained from the DiPs by using the objective lens of NA=0.9. For easy comparison, the equivalent data by using the objective lens of NA=0.9 are displayed in (c) and (d).

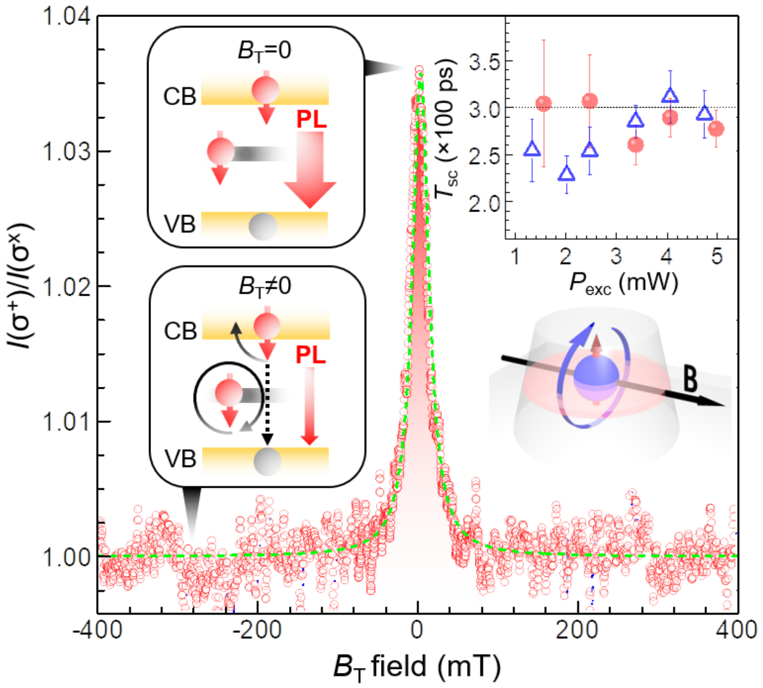

Supplementary Figure 9. Hanle measurement. $I(\sigma^+)/I(\sigma^X)$ ratio (the open circles) as a function of a transverse magnetic field obtained at RT from the Hanle measurements. A strong SDR effect close to zero magnetic field and a weakened SDR effect in a magnetic field due to spin depolarization of the defect electron are illustrated in the inset on the left. The Hanle curve is fitted by a single Lorentzian line (the dashed line). The top-right inset summarizes the power-dependent spin lifetime T_{sc} derived from the Hanle curves from the GaNAs/GaAs DiP (the filled circle) and MQWs (the open triangles). The vertical bars in the insert represent the errors involved in fitting the Hanle curves with a Lorentzian function.

Supplementary Note 1: HH-LH splitting and strain relaxation

The incorporation of N in the GaAs lattice mainly perturbs the conduction band via a mechanism called band anti-crossing between the localized N state and the extended CB minimum, which pushes down the energy of the CB minimum of GaNAs [1,2]. It is commonly accepted that the VB is largely unaffected by N and the offset at the GaNAs/GaAs heterojunction is negligible [3-7]. Therefore, no quantum confinement in the VB and an associated HH-LH splitting are expected in the GaNAs/GaAs QWs.

The HH-LH splitting, commonly observed in GaNAs epilayers and QWs, is caused by the strain field introduced due to the large lattice mismatch between GaAs and GaNAs. The size of the HH-LH splitting is determined by the extent of the lattice mismatch and thus the N composition, which amounts

to 24 - 29 meV over the N composition range of 1.1 % - 1.32 % among the seven GaNAs NDs, based the earlier electroreflectance results from GaNAs epilayers reported by Zhang et al. [8]. Due to the tensile strain experienced by GaNAs, the LH state lies above the HH state in the VB. The resulting PL emission of the band-to-band transitions from GaNAs is therefore composed of the CB-HH and CB-LH transitions with the latter lying at the lower energy. Due to the well-known large potential fluctuations of the CB band edge in GaNAs, the CB-HH and CB-LH PL emissions are not resolved in a PL spectrum. The evidence for their presence can, however, be found from the spectral dependence of PL polarization thanks to the fact that the CB-HH and CB-LH emissions should exhibit opposite helicities of circular polarization. Namely, the CB-HH PL should be co-polarized with the excitation light whereas the CB-LH PL is counter-polarized. In Supplementary Figure 5 we show a typical spectral dependence of the GaNAs MQWs reference sample, which is similar to that from GaNAs epilayers and QWs reported in the literature. Such a spectral dependence is absent in our DiP structure, as shown in Figure 3f of our manuscript, thereby confirming the vanishing HH-LH splitting in the NDs due to strain relaxation. This is consistent with the observed strain relaxation of the GaAs barrier layers in the DiPs shown in Supplementary Figure 6, since the strain fields experienced by the GaNAs NDs and the GaAs barrier layers are mutual and of the same origin – the lattice mismatch between the two materials. We should point out that strain relaxation is a common feature seen in semiconductor NWs/NPs and it has been demonstrated in many material systems.

Supplementary Note 2:

Simulations of detection efficiency and polarization as a function of numerical aperture

To have quantitative insight into NP-tailored light output, we performed simulations of the detection efficiency and polarization as a function of numerical aperture (NA) with the aid of the finite difference time domain (FDTD) calculations by Lumerical's FDTD software. For this purpose, we assumed a spin-polarized dipole emitter located in the middle of the ND stack in the DiP, which also represents the case when the optical effects are averaged over all NDs. The

dipole emission includes recombination of 100% spin-polarized CB electrons with VB HH, LH, and both HH and LH VB holes. In Supplementary Figure 7, PL circular polarization degree, P_{PL}, and detection efficiency, γ, from the DiP and MQW structures are calculated as a function of NA of a microscope objective.

The physical mechanism for the change in P_{PL} of the collected PL from NDs by using objective lenses of different NA stems from scattering and diffraction of the emitting light at the ND surface, e.g. the tapered sidewall. Taking the band-to-band transition associated with the HH state for example, as shown above in Supplementary Figure 7a, the emission light inside NDs is purely polarized in the ND plane (i.e. the *x-y* plane). During transmission through the ND surface, part of the PL light is scattered into polarization along the z-axis perpendicular to the ND plane. When detecting the emission in far-field, this *z*-component contributes to a randomly polarized PL background, which reduces the detected PL circular polarization degree. With a larger NA, the solid angle in light collection by the objective lens increases allowing more scattered *z*-component to be detected. As a result, the PL circular polarization detected in far-field from the NDs rapidly changes with NA. This can also be verified by the CB-LH transition in Supplementary Figure 7b, which readily has an intrinsic *z*-polarized dipole component. Therefore, its polarization change with NA is even more drastic than HH case, which is indeed the case by comparing Supplementary Figure 7a and 7b.

To further justify the conversion factor between the electron spin polarization and PL circular polarization degree, we also performed the same measurements as shown in Figure 3c and 3f of the manuscript, but using an objective lens with a higher NA value of NA=0.9. The results are shown in Supplementary Figure 8a and 8b. The $I(\sigma^{+})/I(\sigma^{X})$ ratio are deduced from Supplementary Figure 8a to be 1.055 (the dashed dotted line) and 1.155 (the dashed line) for from the NDs with $[N_{min}]$=1.1% and $[N_{max}]$=1.32%, respectively. These values are almost identical to that obtained by using an objective lens of NA=0.5, see Supplementary Figure 8c,

which is expected as total PL intensity (including all polarization componentsincluding te scattered light) was detected in measuring the $I(\sigma^{+})/I(\sigma^{X})$ ratio. This finding confirms that identical conditions were employed for both measurements. In sharp contrast, comparing Supplementary Figure 8b with 8d, the PL polarization degree is considerably lower by using an objective lens with a higher NA value of NA=0.9, yielding 5.2% (the dashed dotted line) and 7% (the dashed line) for [N_{min}]=1.1% and [N_{max}]=1.32%, respectively. The decrease of P_{PL} with the larger NA follows the trend predicted by our model in Supplementary Figure 7. Given the conversion factor of 8.3 at NA=0.9 from the simulations, we derive the electron spin polarization to be 58% (43%) for [N_{max}]=1.32% ([N_{min}]=1.1%). This is in good agreement with the corresponding electron spin polarization degree of 60% (43%) obtained by using an objective lens of NA=0.5. The consistency achieved between these two sets of experiments by using different NA values therefore corroborates the accuracy of our analysis and justifies the conversion factor used between the electron spin polarization and PL circular polarization degree.

Supplementary Note 3: Coupled rate equation analysis

For a quantitatively understanding of the SDR effect in the studied DiP structure, we employed a nonlinear coupled rate equation analysis in the form of:

$$\frac{\mathrm{d}n_{\pm}}{\mathrm{d}t} = -\gamma_{\mathrm{e}} n_{\pm} N_{\mp} - \frac{n_{\pm}-n_{\mp}}{2\tau_{\mathrm{s}}} - \frac{n_{\pm}}{\tau_{\mathrm{d}}} + G_{\pm} \quad (1)$$

$$\frac{\mathrm{d}N_{\pm}}{\mathrm{dt}} = -\gamma_{\mathrm{e}} n_{\mp} N_{\pm} - \frac{N_{\pm}-N_{\mp}}{2\tau_{\mathrm{sc}}} + \frac{1}{2}\gamma_{\mathrm{h}} p N_{\uparrow\downarrow} \quad (2)$$

$$\frac{\mathrm{d}p}{\mathrm{dt}} = -\gamma_{\mathrm{h}} p N_{\uparrow\downarrow} + G_{+} + G_{-} - \frac{n_{+}+n_{-}}{\tau_{\mathrm{d}}} \quad (3)$$

$$N_{\mathrm{c}} = N_{\uparrow\downarrow} + N_{+} + N_{-} \quad (4)$$

Here, $G_{\pm}$ is the optical generation rate of CB electrons and VB holes, $n_{\pm}(N_{\pm})$ represents the concentration of CB electrons (concentration of the $\mathrm{Ga_i}$ defect in the paramagnetic charge state

occupied by a single electron) where the subscript '±' denotes the spin-up and spin-down orientation of electrons with $S_z = \pm 1/2$. $N_{\uparrow\downarrow}$ is the concentration of the defect in the charge state occupied by two spin-paired electrons and N_c defines the total defect concentration. The concentration of VB holes is denoted by p. $\tau_s(\tau_{sc})$ is the spin relaxation time of CB (defect) electrons. τ_d is free carrier lifetime accounting for both radiative and other non-radiative recombination except the Ga_i defects. $\gamma_e(\gamma_h)$ is the trapping coefficient of CB electrons (VB holes) by the Ga_i defect, with γ_e/γ_h = 4 that is known from early studies and is an intrinsic property of a given defect irrespective of N content [9]. We use the reported value of τ_s = 150 ps from Refs. 2 and 3 and τ_{sc} = 300 ps determined from our Hanle measurements (see Supplementary Note 4 below and Supplementary Figure 9). τ_d takes a shorter lifetime of 3 ns than the reported value of 10 ns in GaNAs thin films and MQWs [9, 10], in view of the aforementioned three-times lower radiative efficiency of the NDs due to surface recombination. Here, only $\gamma_e N_c$ is a fitting parameter. The best fits to the power-dependent P_e profiles shown by the solid lines in Figure 4 yield $\gamma_e N_c$ = 0.4383 ps^{-1} (0.7012 ps^{-1}) for [N_{min}] ([N_{max}]). The higher $\gamma_e N_c$ for [N_{max}] than [N_{min}] is reasonable, since the NDs with [N_{max}] contains more defects. Assuming a value of γ_e commonly found for a deep-level defect in GaAs, i.e. γ_e =1.7×10^{-16} cm^3/ps, we can estimate the concentration of the Ga_i defect to be approximately 2.6(±0.2)×10^{15} cm^{-3} and 4.1(±0.5)×10^{15} cm^{-3} in the NDs with [N_{min}] and [N_{max}], respectively. This corresponds to 2 defects in each ND with [N_{min}] and 3 defects in each ND with [N_{max}]. The same set of rate equations was also used in the calculations of electron spin polarization and internal quantum efficiency shown in Figure 5 of main text.

For comparison, we also performed calculations of electron spin polarization and internal quantum efficiency in the presence of a defect without the SDR effect. The results are shown in Figure 5. Here, the available charge transition level of the defect is assumed to correspond to a transition between the charge states with zero and one electron, such that capture of CB

electrons by the defect with zero electron is spin independent. A suitable set of rate equations is as follows:

$$\frac{\mathrm{d}n_\pm}{\mathrm{d}t} = -\gamma_\mathrm{e} n_\pm N_0 - \frac{n_\pm - n_\mp}{2\tau_\mathrm{s}} - \frac{n_\pm}{\tau_\mathrm{r}} + G_\pm \quad (5)$$

$$\frac{\mathrm{d}N_0}{\mathrm{d}t} = -\gamma_\mathrm{e}(n_+ + n_-)N_0 + \gamma_\mathrm{h} p N_1 \quad (6)$$

$$\frac{\mathrm{d}p}{\mathrm{dt}} = -\gamma_\mathrm{h} p N_1 + G_+ + G_- - \frac{n_+ + n_-}{\tau_\mathrm{r}} \quad (7)$$

$$N_\mathrm{c} = N_0 + N_1 \quad (8)$$

Here, N_0 and N_1 represent the density of the defect with 0 and 1 electron. The rate equations can be reduced to the well-known simple equation

$$P_\mathrm{e} = \frac{n_+ - n_-}{n_+ + n_-} = \frac{P_0}{1+\frac{\tau}{\tau_\mathrm{S}}} \quad (9)$$

where $\frac{1}{\tau} = \frac{1}{\tau_\mathrm{r}} + \gamma_\mathrm{e} N_0$. It should be noted that, strictly speaking, N_0 and thus τ are no longer constants here. They will approximately be constants only under the condition when the defects are predominantly in the charge state with no electron, i.e. $N_0 \approx N_\mathrm{c}$.

Supplementary Note 4: Room-temperature Hanle measurements

In Hanle measurements, a transverse magnetic field, $\mathrm{B_T}$, was applied in the direction perpendicular to the direction of the optically oriented electron spins that coincides with the NP axis as schematically illustrated in Supplementary Figure 9. The spin blockade in capture of CB electrons by the defects, which is responsible for the observed non-zero SDR ratio and strong electron spin polarization at zero field, breaks down upon the application of $\mathrm{B_T}$ due to the Larmor precession of the defect electron spins. This activates the capture and recombination via the defects, leading to a decrease in the BB PL intensity and thus in the SDR ratio. Therefore, the field dependence of the SDR ratio should be directly correlated with the spin depolarization of the $\mathrm{Ga_i}$ defects, which can be described by a Hanle curve of a Lorentzian lineshape [11].

Indeed, the measured Hanle curve by monitoring the ND PL at 1000 nm under P_{exc} of 5 mW is dominated by a sharp Lorentzian peak, as shown by the open circles in Supplementary Figure 9. The half width at half maximum (HWHM), $B_{1/2}$, is determined by the spin lifetime of the defect responsible for the SDR, T_{sc}, i.e., $g_{\mathrm{e}}T_{\mathrm{sc}} = \frac{\hbar}{\mu_{\mathrm{B}}B_{1/2}}$ [11]. Here, g_{e} is the Landé g-factor of the defect with a typical value of 2 [9], $\hbar$ is the reduced Plank constant, and μ_{B} is the Bohr magneton. From the best fit to the Hanle curve, T_{sc} of ~280 ps can be determined. T_{sc} is governed by the lifetime of the defect in its paramagnetic charge state, τ, and the spin relaxation time, τ_{sc}, i.e., $T_{\mathrm{sc}}^{-1} = \tau^{-1} + \tau_{\mathrm{sc}}^{-1}$ [11]. Approaching the low excitation limit where defect capture time becomes exceedingly long, i.e., $\tau \gg \tau_{\mathrm{sc}}$, τ_{sc} approaches T_{sc}. The power dependence of T_{sc} is presented in the top-right inset of Supplementary Figure 9 by the filled circles, which shows a stable low-power limit of $\tau_{\mathrm{sc}} \approx 300$ ps. This value is approximately the same as that determined from the unetched reference MQW sample, see the open triangles in the top-right inset of Supplementary Figure 6, which rules out a potential surface effect on τ_{sc}, e.g. via spin-orbit interaction promoted by a surface electric field. This is not surprising as Ga_i is a point defect with a highly localized electron wavefunction such that its electronic and spin properties are predominantly governed by the local potential, making τ_{sc} insusceptible to a surface field.

Supplementary References

1. Buyanova, I. A. & Chen, W. M. Physics and applications of dilute nitrides (Taylor & Francis, New York, 2004).
2. Shan, W. *et al.* Band anticrossing in GaInNAs alloys. *Phys. Rev. Lett.* **82**, 1221-1224 (1999).
3. Kitani T., Kondow M., Kikawa T. Yazawa Y., Okai M. & Uomi K. Analysis of band offset in GaNAs/GaAs by X-ray photoemission spectroscopy. *Jpn. J. Appl. Phys.* **38**, 5003-5006 (1999).

4. Shan, W., Walukiewicz, W., Ager III, J. W. & Haller, E. E., Geisz J. F., Friedman D. J., Olson J. M. & Kurtz S. R. Effect of nitrogen on the band structure of GaInNAs alloys. *J. Appl. Phys*. **86**, 2349-2351 (1999).

5. Shan, W., Yu, K. M., Walukiewicz, W., Wu, J., Ager III, J. W. & Haller, E. E. Band anticrossing in dilute nitrides. *J. Phys.: Condens. Matter* **16**, S3355 – S3372 (2004).

6. Skierbiszewski, C., Perlin P., Wisniewski P., Suski T., Geisz J. F., Hingerl K., Jantsch W., Mars D. E., & Walukiewicz, W. Band structure and optical properties of $In_yGa_{1-y}As_{1-x}N_x$ alloys. *Phys. Rev. B* **65**, 035207 (2001).

7. Krispin P., Spruytte S.G., Harris J. S. & Ploog K. H. Admittance dispersion of n-type GaAs/Ga(As,N)/GaAs heterostructure grown by molecular beam epitaxy. *J. Appl. Phys.* **90**, 2405-2410 (2001).

8. Zhang, Y., Mascarenhas, A., Xin, H. P. & Tu, C. W. Valence-band splitting and shear deformation potential of dilute GaAs1-xNx alloys. *Phys. Rev. B* **61**, 4433 (2000).

9. Wang, X. J. *et al.* Room-temperature defect-engineered spin filter based on a non-magnetic semiconductor. *Nat. Mater*. **8**, 198 – 202 (2009).

10. Lagarde, D. *et al*. Electron spin dynamics in GaAsN and InGaAsN structures. *Phys. stat. sol. (a)* **204**, 208 - 220 (2007).

11. Dyakonov, M. I. Spin physics in semiconductors (Springer, Berlin, Heidelberg, 2008).
